# Crystal structure of Cr-bearing Mg_3_BeAl_8_O_16_, a new polytype of magnesiotaaffeite-2*N*′2*S*

**DOI:** 10.1107/S2056989016010215

**Published:** 2016-06-28

**Authors:** Thomas Malcherek, Jochen Schlüter

**Affiliations:** a Mineralogisch-Petrographisches Institut, Universität Hamburg, Grindelallee 48, 20146 Hamburg, Germany; b Centrum für Naturkunde (CeNak), Mineralogisches Museum, Universität Hamburg, Grindelallee 48, 20146 Hamburg, Germany

**Keywords:** crystal structure, magnesiotaaffeite, gemstones, polytypism, polysomatism, modular structure

## Abstract

The crystal structure of a new polytype of magnesiotaaffeite-2*N*′2*S*, ideally Mg_3_BeAl_8_O_16_ (trimagnesium beryllium octa­aluminium hexa­deca­oxide), is described in space-group symmetry *P*-3*m*1. It has been identified in a fragment of a mineral sample from Burma (Myanmar). The new polytype is composed of two Mg_2_Al_4_O_8_ (*S*)- and two BeMgAl_4_O_8_ (*N*′)-modules in a stacking sequence *N*′*SSN*′′ which differs from the *N*′*SN*′*S*-stacking sequence of the known magnesiotaaffeite-2*N*′2*S* polytype. The crystal structure can be derived from a close-packed arrangement of O atoms and is discussed with regard to its polytypism and its Cr^3+^ chromophore content.

## Abstract 2

The crystal structure of a new polytype of magnesiotaaffeite-2*N′*2*S* is described. The *S* (Mg_2_Al_4_O_8_) and *N*′ (BeMgAl_4_O_8_) modules have the stacking sequence *N*′*SSN*′′.

## Mineralogical and crystal-chemical context   

The minerals of the taaffeite group form a polysomatic series, composed of spinel (*S*) and nolanite (*N*′) modules (Armbruster, 2002 ▸). The nolanite modules in the taaffeites are modified with respect to the nolanite, (V,Fe)_5_O_7_(OH), crystal structure (Gatehouse *et al.*, 1983 ▸), such that Be nominally substitutes for the hydrogen atoms of the nolanite OH group, while Mg and Al replace V and Fe, respectively. Variable numbers of the *S*-modules, Mg_2_Al_4_O_8_, and of the *N*′-modules, BeMgAl_4_O_8_, combine to yield different compositions of taaffeite minerals, *i.e.* different polysomes. Magnesiotaaffeite-2*N*′2*S* is composed of two modified nolanite modules *N*′ and two spinel modules *S*, yielding an idealized composition of Mg_3_BeAl_8_O_16_. Be-doping of MgAl_2_O_4_ has been shown to cause growth of twinned spinel crystals as a precursor to the formation of magnesiotaaffeite polytypes (Drev *et al.*, 2013 ▸).

Here we report the crystal structure of a new polytype of magnesiotaaffeite, magnesiotaaffeite-2*N′*2*S*_2_ which differs from the known magnesiotaaffeite-2*N*′2*S* (Nuber & Schmetzer, 1983 ▸) by the module stacking sequence. The resulting space group symmetry is *P*-3*m*1, as opposed to the *P*6_3_ *mc* symmetry of the previously known polytype.

## Structural commentary   

**Figure 1:**
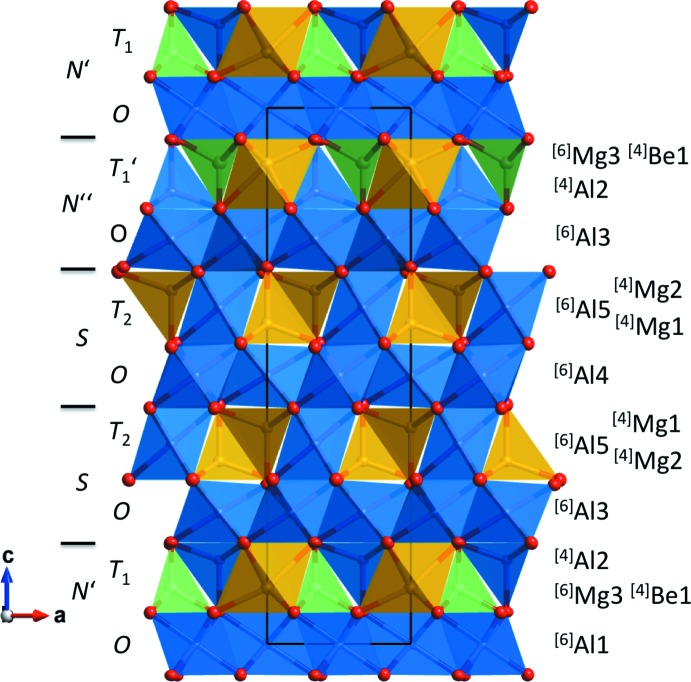
Polyhedral plot of magnesiotaaffeite-2*N*′2*S*_2_ viewed down [010] with cation site nomenclature and coordination numbers given to the right. Module sequence is *N*′–*S*–*S*–*N*′′–*N*′ from bottom to top, with boundaries indicated by horizontal lines. Displacement ellipsoids are drawn at the 99% probability level. Mg atoms are shown in yellow, Al in blue, Be in green and O in red.

The crystal structure of the title compound is shown in Fig. 1 ▸. It can be described by the stacking of close-packed oxygen layers along [001], with layers of cations filling the inter­stices. Following the layer nomenclature of Nuber & Schmetzer (1983 ▸), the ^[6]^Al1, ^[6]^Al3 and ^[6]^Al4 cations can be attributed to *O*-layers, the ^[6]^Al5, ^[4]^Mg1 and ^[4]^Mg2 cations to *T*_2_-layers and the ^[4]^Be,^[4]^Al2 and ^[6]^Mg3 cations to *T*_1_-layers. The cation stacking sequence is then *T*_1_-*O*-*T*_2_-*O*-*T*_2_-*O*-*T*_1_′-⋯ while the anion stacking sequence is *BACBACBC*⋯. The orientation of *T*_1_′ is upside down with respect to *T*_1_. In the polytype described by Nuber & Schmetzer (1983 ▸), the stacking sequence is *T*_1_-*O*-*T*_2_-*O*-*T*_1_-*O*-*T*_2_-*O*-⋯ and *BCABCBAC*⋯ by comparison. In terms of polysomatism, the *N*′ layer is composed of one *T*_1_ and one *O*-layer. The second nolanite layer, *N*′′, is also composed of these layer types, but its *T*_1_ layer is inverted with respect to the stacking direction. The *S*-layer is composed of one *O*-layer and one *T*_2_-layer. Stacking these modules in the order *N*′-*S*-*S*-*N*′′-*N*′-⋯ generates the new polytype structure (Fig. 1 ▸). The stacking sequence of the known magnesiotaaffeite-2*N*′2*S* polytype is *N*′-*S*-*N*′-*S*- ⋯.

The composition obtained by structure refinement is in good agreement with the composition obtained by electron microprobe analysis (EMPA). The calculated bond-valence sums agree reasonably well with the formal charges (Table 1 ▸), and on average they support the assumption that Cr is trivalent. Significant amounts of Cr^3+^ are found at the octa­hedrally coordinated Al3 and Al4-sites, where Cr^3+^ is overbonded, as well as at the tetra­hedrally coordinated Mg1 and Mg2 sites, where Cr^3+^ is underbonded. Cr^3+^ in tetra­hedral coordination is unusual, but has recently been reported for the brownmiller­ite-type compound Ca_2_Cr_2_O_5_ (Arevalo-Lopez & Attfield, 2015 ▸) and for Cr-doped BaAl_2_O_4_ (Vrankić *et al.*, 2015 ▸). However, without further confirmation by other methods, the appearance of tetra­hedrally coordinated Cr^3+^ in the title compound should be treated with caution. The tetra­hedral Mg1 coordination, with one Mg1—O6 distance of 1.9537 (12) Å and three Mg1—O4 distances of 1.9296 (7) Å is more distorted than the Mg2 coordination environment, where the longer Mg2—O1 distance [1.9361 (13) Å] hardly differs from the three 1.9300 (7) Å Mg2—O5 distances. The average bond lengths at the tetra­hedral sites, nominally occupied by Mg (Mg1 1.936 Å, Mg2 1.932 Å), and at the octa­hedral sites, nominally occupied by Al (Al1 1.909 Å, Al3 1.916 Å, Al4 1.913 Å, Al5 1.909 Å), are similar to the *T*-O (1.936 Å) and *M*-O (1.923 Å) distances reported for a natural Cr and V-bearing spinel from Burma with a small inversion parameter (Widmer *et al.*, 2015 ▸). This indicates that the degree of Mg, Al disorder is equally low in the title compound. The Al2 site is at the center of a nearly regular oxygen tetra­hedron with an average Al—O distance of 1.785 Å. Al^3+^ is slightly underbonded at this site (Table 1 ▸), which might indicate admixture of Mg atoms. The slightly overbonded Mg2 site might accommodate the resulting Al-excess. The Be^2+^ cation forms one short bond with O7 [1.602 (2) Å] and three longer bonds [1.6615 (13) Å] with the O3-anions, while the tetra­hedral angles are either 97.89 (9)° (O3—Be1—O3) or 119.45 (7)° (O7—Be1—O3). The Mg atom in the Mg3O_6_-octa­hedron exhibits a strong out-of-centre distortion, away from the Al3-cation, to which it has a distance of only 3.0580 (7) Å.

Rotation of the refined crystal structure by 60° about [001] brings the bottom *O*-layer (Fig. 1 ▸) into the same orientation as the third *O*-layer of the unrotated structure. Thus a corres­pondingly rotated twin domain of the polytype structure can form a strain free boundary after the first *S*-layer of the module sequence as shown in Fig. 1 ▸. At the twin boundary this results in a module sequence *N*′-*S*-*N*′-*S*, corresponding to the previously described polytype.

## Sample details and EMPA   

**Figure 2:**
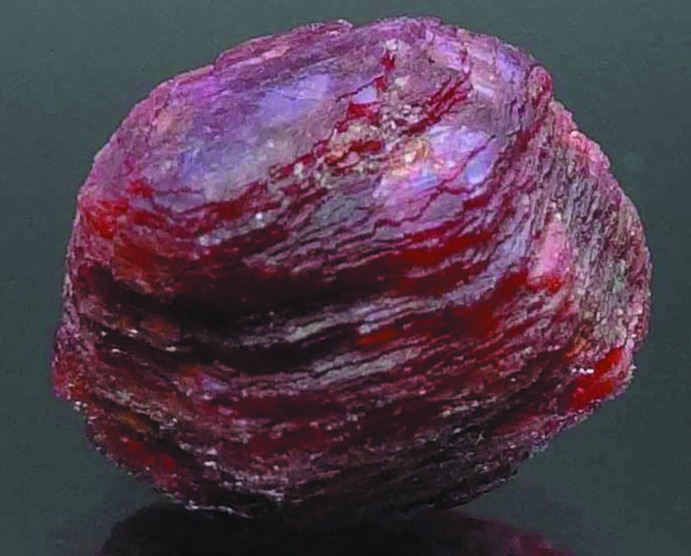
Magnesiotaaffeite sample, approximate size 1.0 × 0.9 × 0.8 cm. (Photograph courtesy of Daniela Braith, Munich.)

The studied natural sample of magnesiotaaffeite (*m* = 0.95 g) originates from Chaung-gyi, Mogok, Pyin-Oo-Lwin district, Burma (Myanmar). It has a red colour and a layered appearance (Fig. 2 ▸). A small fragment of the original sample was examined using single crystal X-ray diffraction. The same crystal fragment was subsequently prepared for electron microprobe analysis (EMPA) using a Cameca SX100 electron microprobe, operating in wavelength-dispersion mode at 15 kV and 20 nA. Standards were MgO, Al_2_O_3_ and Cr_2_O_3_. Based on 16 anions and the Be concentration from single-crystal X-ray structure refinement (1 Be), the empirical chemical formula was determined as Al_7.86_Be_1.0_Cr_0.19_Mg_2.93_O_16_. The corresponding oxide composition (in wt%) is MgO 20.82, Cr_2_O_3_ 2.50, Al_2_O_3_ 70.72, BeO 4.41, yielding a total of 98.45%.

## Refinement   

Crystal data, data collection and structure refinement details are summarized in Table 2 ▸. The results of the EMPA indicate that the magnesiotaaffeite crystal contains significant amounts of Cr. In order to accurately refine small Cr-site populations against the major constituent elements Al and Mg, intensities at small scattering angles were systematically weighted down by a factor of 1−exp[−5(sin θ/λ)^2^] in order to emphasize core electron contributions to the X-ray scattering. For that purpose, Cr and Mg or Al were constrained to have the same coordinates and displacement parameters under consideration of full occupancy for the corresponding site. Scattering factors for neutral atoms were used and all atoms were refined with anisotropic displacement parameters. No evidence for mixed occupancy was found at the Be site; small Cr amounts were found for the Al3, Al4, Mg1 and Mg2 sites with occupation factors for Cr of 0.017 (3), 0.017 (5), 0.028 (5) and 0.048 (5), respectively. Two twin domains (twinning by merohedry) with volume fractions of 0.64 and 0.36 contribute to the total scattering intensity, related by reflection parallel to [1
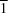
0] or, equivalently, by 60° rotation about [001].

## Supplementary Material

Crystal structure: contains datablock(s) I. DOI: 10.1107/S2056989016010215/wm5296sup1.cif

Structure factors: contains datablock(s) I. DOI: 10.1107/S2056989016010215/wm5296Isup2.hkl

CCDC reference: 1487140

Additional supporting information: crystallographic information; 3D view; checkCIF report

## Figures and Tables

**Table 1:** Bond-valence sums (BVS) Calculated using *JANA2006* (Petřìček *et al.*, 2014 ▸) with bond-valence parameters taken from Brese & O’Keeffe (1991 ▸). Angular brackets indicate site-occupancy weighted averages for the corresponding *Mab* sites.

Site	BVS
Be1	1.956 (5)
Al1	3.007 (2)
Al2	2.788 (3)
Al3*a*	2.932 (2)
Cr3*b*	3.571 (3)
<*M*3*ab*>	2.943
Al4*a*	2.955 (2)
Cr4*b*	3.599 (3)
<*M*4*ab*>	2.966
Al5	2.991 (2)
Mg1*a*	2.077 (2)
Cr1*b*	2.259 (2)
<*M*1*ab*>	2.082
Mg2*a*	2.099 (3)
Cr2*b*	2.283 (3)
<*M*2*ab*>	2.108
Mg3	1.974 (2)
O1	2.006 (2)
O2	1.991 (3)
O3	1.962 (2)
O4	2.009 (2)
O5	2.008 (2)
O6	2.045 (2)
O7	1.993 (4)
O8	1.906 (2)

**Table 2:** Experimental details

Crystal data	
Chemical formula	Mg_3_BeAl_8_O_16_
*M*_r_	557.75
Crystal system, space group	Trigonal, *P* 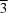 *m*1
Temperature (K)	295
*a*, *c* (Å)	5.6788 (3), 18.3368 (14)
*V* (Å^3^)	512.11 (7)
*Z*	2
Radiation type	Mo *K*α
μ (mm^−1^)	1.25
Crystal size (mm)	0.23 × 0.22 × 0.10

Data collection	
Diffractometer	Nonius KappaCCD
Absorption correction	Multi-scan (*SADABS*; Bruker, 2008 ▸)
*T*_min_, *T*_max_	0.614, 0.747
No. of measured, independent and observed [*I* > 2σ(*I*)] reflections	10618, 940, 912
*R*_int_	0.040
(sin θ/λ)_max_ (Å^−1^)	0.807

Refinement	
*R*[*F* ^2^ > 2σ(*F* ^2^)], *wR*(*F* ^2^), *S*	0.018, 0.040, 0.83
No. of reflections	940
No. of parameters	75
Δρ_max_, Δρ_min_ (e Å^−3^)	0.41, −1.01

Computer programs: *COLLECT* (Nonius, 1998 ▸), *EVAL15*/*Peakref* and *EVAL15* (Schreurs *et al.*, 2010 ▸), *SUPERFLIP* (Palatinus & Chapuis, 2007 ▸), *SHELXL2014* (Sheldrick, 2015 ▸), *VESTA* (Momma & Izumi, 2011 ▸) and *publCIF* (Westrip, 2010 ▸).

## supplementary crystallographic information

### Crystal data

**Table:**

Mg_3_BeAl_8_O_16_	*D*_x_ = 3.617 Mg m^−^^3^
*M**_r_* = 557.75	Mo *K*α radiation, λ = 0.71069 Å
Trigonal, *P*3*m*1	Cell parameters from 9717 reflections
*a* = 5.6788 (3) Å	θ = 2.2–35°
*c* = 18.3368 (14) Å	µ = 1.25 mm^−^^1^
*V* = 512.11 (7) Å^3^	*T* = 295 K
*Z* = 2	Tabular, red
*F*(000) = 547	0.23 × 0.22 × 0.10 mm

### Data collection

**Table:**

Nonius KappaCCD diffractometer	940 independent reflections
Radiation source: fine-focus sealed X-ray tube	912 reflections with *I* > 2σ(*I*)
Graphite monochromator	*R*_int_ = 0.040
Detector resolution: 9 pixels mm^-1^	θ_max_ = 35.0°, θ_min_ = 2.2°
φ scans, and ω scans with κ offsets	*h* = −8→9
Absorption correction: multi-scan (*SADABS*; Bruker, 2008)	*k* = −9→8
*T*_min_ = 0.614, *T*_max_ = 0.747	*l* = −29→29
10618 measured reflections	

### Refinement

**Table:**

Refinement on *F*^2^	0 restraints
Least-squares matrix: full	*w* = {1-exp[-5(sinθ/λ)^2^)]}/[σ^2^(*F*_o_^2^) + (0.0296*P*)^2^ + 0.031*P*] where *P* = (*F*_o_^2^ + 2*F*_c_^2^)/3
*R*[*F*^2^ > 2σ(*F*^2^)] = 0.018	(Δ/σ)_max_ < 0.001
*wR*(*F*^2^) = 0.040	Δρ_max_ = 0.41 e Å^−^^3^
*S* = 0.83	Δρ_min_ = −1.01 e Å^−^^3^
940 reflections	Extinction correction: SHELXL2014 (Sheldrick, 2015), Fc^*^=kFc[1+0.001xFc^2^λ^3^/sin(2θ)]^-1/4^
75 parameters	Extinction coefficient: 0.046 (3)

### Special details

**Table:**

Geometry. All esds (except the esd in the dihedral angle between two l.s. planes) are estimated using the full covariance matrix. The cell esds are taken into account individually in the estimation of esds in distances, angles and torsion angles; correlations between esds in cell parameters are only used when they are defined by crystal symmetry. An approximate (isotropic) treatment of cell esds is used for estimating esds involving l.s. planes.
Refinement. Refined as a 2-component twin

### Fractional atomic coordinates and isotropic or equivalent isotropic displacement parameters (Å^2^)

**Table:**

	*x*	*y*	*z*	*U*_iso_*/*U*_eq_	Occ. (<1)
Be1	0.3333	0.6667	0.10240 (12)	0.0054 (3)	
Al1	0.5000	0.0000	0.0000	0.00335 (9)	
Al2	0.6667	0.3333	0.15613 (3)	0.00344 (9)	
Al3A	−0.33348 (5)	−0.16674 (3)	0.24468 (2)	0.00340 (11)	0.983 (3)
Cr3B	−0.33348 (5)	−0.16674 (3)	0.24468 (2)	0.00340 (11)	0.017 (3)
Al4A	0.5000	0.0000	0.5000	0.00331 (15)	0.983 (5)
Cr4B	0.5000	0.0000	0.5000	0.00331 (15)	0.017 (5)
Al5	0.3333	0.6667	0.37276 (3)	0.00293 (9)	
Mg1A	0.0000	0.0000	0.40321 (3)	0.0040 (2)	0.972 (5)
Cr1B	0.0000	0.0000	0.40321 (3)	0.0040 (2)	0.028 (5)
Mg2A	0.6667	0.3333	0.34070 (3)	0.0041 (2)	0.952 (5)
Cr2B	0.6667	0.3333	0.34070 (3)	0.0041 (2)	0.048 (5)
Mg3	0.0000	0.0000	0.10393 (4)	0.00473 (11)	
O1	0.6667	0.3333	0.44628 (7)	0.00471 (17)	
O2	0.6667	0.3333	0.05925 (6)	0.00435 (18)	
O3	−0.81376 (7)	−0.18624 (7)	0.05785 (4)	0.00439 (12)	
O4	−0.81598 (7)	−0.18402 (7)	0.43966 (4)	0.00449 (12)	
O5	−0.51854 (7)	−0.03709 (14)	0.30594 (3)	0.00497 (12)	
O6	0.0000	0.0000	0.29666 (6)	0.00458 (18)	
O7	0.3333	0.6667	0.18977 (6)	0.00459 (18)	
O8	−0.16175 (6)	−0.83825 (6)	0.18822 (4)	0.00461 (11)	

### Atomic displacement parameters (Å^2^)

**Table:**

	*U*^11^	*U*^22^	*U*^33^	*U*^12^	*U*^13^	*U*^23^
Be1	0.0056 (5)	0.0056 (5)	0.0050 (7)	0.0028 (2)	0.000	0.000
Al1	0.00339 (12)	0.00316 (15)	0.00343 (15)	0.00158 (7)	0.00049 (6)	0.00098 (11)
Al2	0.00375 (12)	0.00375 (12)	0.00281 (18)	0.00188 (6)	0.000	0.000
Al3A	0.00313 (13)	0.00356 (12)	0.00337 (14)	0.00157 (7)	0.00005 (8)	0.00003 (4)
Cr3B	0.00313 (13)	0.00356 (12)	0.00337 (14)	0.00157 (7)	0.00005 (8)	0.00003 (4)
Al4A	0.00349 (17)	0.00302 (19)	0.00326 (19)	0.00151 (10)	0.00000 (5)	0.00000 (11)
Cr4B	0.00349 (17)	0.00302 (19)	0.00326 (19)	0.00151 (10)	0.00000 (5)	0.00000 (11)
Al5	0.00329 (12)	0.00329 (12)	0.00220 (16)	0.00165 (6)	0.000	0.000
Mg1A	0.0041 (2)	0.0041 (2)	0.0039 (3)	0.00204 (12)	0.000	0.000
Cr1B	0.0041 (2)	0.0041 (2)	0.0039 (3)	0.00204 (12)	0.000	0.000
Mg2A	0.0043 (2)	0.0043 (2)	0.0038 (3)	0.00215 (11)	0.000	0.000
Cr2B	0.0043 (2)	0.0043 (2)	0.0038 (3)	0.00215 (11)	0.000	0.000
Mg3	0.00494 (15)	0.00494 (15)	0.0043 (2)	0.00247 (8)	0.000	0.000
O1	0.0045 (2)	0.0045 (2)	0.0051 (4)	0.00226 (12)	0.000	0.000
O2	0.0048 (2)	0.0048 (2)	0.0034 (4)	0.00241 (12)	0.000	0.000
O3	0.00421 (19)	0.00421 (19)	0.0045 (3)	0.0019 (2)	0.00005 (9)	−0.00005 (9)
O4	0.00431 (19)	0.00431 (19)	0.0049 (3)	0.0022 (2)	−0.00011 (9)	0.00011 (9)
O5	0.00519 (19)	0.0047 (2)	0.0049 (3)	0.00234 (12)	0.00010 (9)	0.00020 (18)
O6	0.0049 (3)	0.0049 (3)	0.0039 (4)	0.00247 (13)	0.000	0.000
O7	0.0053 (3)	0.0053 (3)	0.0031 (4)	0.00267 (13)	0.000	0.000
O8	0.00479 (18)	0.00479 (18)	0.0048 (2)	0.0028 (2)	−0.00033 (10)	0.00033 (10)

### Geometric parameters (Å, º)

**Table:**

Be1—O7	1.602 (2)	Mg1A—Al5^xiii^	3.3259 (2)
Be1—O3^i^	1.6615 (13)	Mg1A—Al3A^iv^	3.3376 (6)
Be1—O3^ii^	1.6615 (13)	Mg1A—Cr3B^iv^	3.3376 (6)
Be1—O3^iii^	1.6615 (13)	Mg1A—Al3A^iii^	3.3376 (6)
Be1—Al1^iv^	2.4926 (17)	Mg1A—Cr3B^iii^	3.3376 (6)
Be1—Al1^v^	2.4926 (17)	Mg2A—O5^viii^	1.9300 (7)
Be1—Al1^vi^	2.4926 (17)	Mg2A—O5^xxiv^	1.9300 (7)
Al1—O3^vii^	1.8799 (5)	Mg2A—O5^iii^	1.9300 (7)
Al1—O3^viii^	1.8799 (5)	Mg2A—O1	1.9361 (13)
Al1—O3^ix^	1.8799 (5)	Mg2A—Al5^viii^	3.3310 (2)
Al1—O3^x^	1.8799 (5)	Mg2A—Al5^xiii^	3.3310 (2)
Al1—O2	1.9666 (6)	Mg2A—Al3A^iv^	3.3410 (3)
Al1—O2^xi^	1.9667 (6)	Mg2A—Al3A^viii^	3.3410 (3)
Al1—Be1^xii^	2.4926 (17)	Mg2A—Al3A^xvi^	3.3410 (3)
Al1—Be1^xiii^	2.4926 (17)	Mg2A—Al3A^xxiv^	3.3410 (3)
Al1—Al1^xiv^	2.8394 (2)	Mg2A—Al3A^iii^	3.3410 (3)
Al1—Al1^xv^	2.8394 (1)	Mg3—O3^xvii^	2.0173 (7)
Al1—Al1^vi^	2.8394 (2)	Mg3—O3^ii^	2.0173 (7)
Al1—Al1^xvi^	2.8394 (1)	Mg3—O3^viii^	2.0173 (7)
Al2—O2	1.7765 (12)	Mg3—O8^xvi^	2.2181 (8)
Al2—O8^i^	1.7874 (7)	Mg3—O8^v^	2.2181 (8)
Al2—O8^iv^	1.7874 (7)	Mg3—O8^xviii^	2.2181 (8)
Al2—O8^xvi^	1.7874 (7)	Mg3—Al3A^iii^	3.0580 (7)
Al3A—O6	1.8969 (6)	Mg3—Cr3B^iii^	3.0580 (7)
Al3A—O5^xvii^	1.9189 (5)	Mg3—Al3A^iv^	3.0580 (7)
Al3A—O5	1.9189 (5)	Mg3—Cr3B^iv^	3.0580 (7)
Al3A—O8^v^	1.9193 (5)	O1—Cr4B^xv^	1.9125 (6)
Al3A—O8^xviii^	1.9193 (5)	O1—Al4A^xv^	1.9125 (6)
Al3A—O7^xix^	1.9232 (6)	O1—Cr4B^vi^	1.9125 (6)
Al3A—Cr3B^xvii^	2.8381 (5)	O1—Al4A^vi^	1.9125 (6)
Al3A—Al3A^xvii^	2.8381 (5)	O2—Al1^xv^	1.9666 (6)
Al3A—Al3A^xx^	2.8381 (5)	O2—Al1^vi^	1.9666 (6)
Al3A—Cr3B^xx^	2.8381 (5)	O3—Be1^xix^	1.6616 (13)
Al3A—Cr3B^iii^	2.8407 (5)	O3—Al1^xviii^	1.8799 (5)
Al3A—Al3A^iii^	2.8407 (5)	O3—Al1^xxv^	1.8799 (5)
Al4A—O1^xxi^	1.9124 (6)	O3—Mg3^xxv^	2.0173 (7)
Al4A—O1	1.9125 (6)	O4—Cr4B^xxv^	1.9133 (5)
Al4A—O4^xxii^	1.9133 (5)	O4—Al4A^xxv^	1.9133 (5)
Al4A—O4^x^	1.9133 (5)	O4—Cr4B^xviii^	1.9133 (5)
Al4A—O4^xxiii^	1.9133 (5)	O4—Al4A^xviii^	1.9133 (5)
Al4A—O4^viii^	1.9133 (5)	O4—Al5^xix^	1.9136 (8)
Al4A—Al4A^xiv^	2.8394 (1)	O4—Cr1B^xxv^	1.9295 (7)
Al4A—Al4A^xv^	2.8394 (1)	O4—Mg1A^xxv^	1.9295 (7)
Al4A—Cr4B^xiv^	2.8394 (1)	O5—Al5^xix^	1.9036 (7)
Al4A—Cr4B^xv^	2.8394 (1)	O5—Cr3B^xx^	1.9188 (5)
Al4A—Al4A^vi^	2.8394 (1)	O5—Al3A^xx^	1.9188 (5)
Al4A—Cr4B^vi^	2.8394 (1)	O5—Cr2B^xxv^	1.9300 (7)
Al5—O5^iii^	1.9037 (7)	O5—Mg2A^xxv^	1.9300 (7)
Al5—O5^ii^	1.9037 (7)	O6—Cr3B^iii^	1.8969 (6)
Al5—O5^i^	1.9037 (7)	O6—Al3A^iii^	1.8969 (6)
Al5—O4^iii^	1.9136 (8)	O6—Cr3B^iv^	1.8969 (6)
Al5—O4^ii^	1.9136 (8)	O6—Al3A^iv^	1.8969 (6)
Al5—O4^i^	1.9136 (8)	O7—Cr3B^ii^	1.9232 (6)
Al5—Cr4B^v^	2.8515 (4)	O7—Al3A^ii^	1.9232 (6)
Al5—Al4A^v^	2.8515 (4)	O7—Al3A^i^	1.9232 (6)
Al5—Cr4B^iv^	2.8515 (4)	O7—Cr3B^i^	1.9232 (6)
Al5—Al4A^iv^	2.8515 (4)	O7—Cr3B^iii^	1.9232 (6)
Al5—Cr4B^vi^	2.8515 (4)	O7—Al3A^iii^	1.9232 (6)
Al5—Al4A^vi^	2.8515 (4)	O8—Al2^xix^	1.7874 (7)
Mg1A—O4^viii^	1.9295 (7)	O8—Cr3B^x^	1.9193 (5)
Mg1A—O4^ii^	1.9296 (7)	O8—Al3A^x^	1.9193 (5)
Mg1A—O4^xvii^	1.9296 (7)	O8—Al3A^xiii^	1.9193 (5)
Mg1A—O6	1.9537 (12)	O8—Cr3B^xiii^	1.9193 (5)
Mg1A—Al5^xix^	3.3259 (2)	O8—Mg3^xiii^	2.2181 (8)
			
O7—Be1—O3^i^	119.45 (7)	O4^viii^—Mg1A—O4^ii^	108.66 (2)
O7—Be1—O3^ii^	119.45 (7)	O4^viii^—Mg1A—O4^xvii^	108.66 (2)
O3^i^—Be1—O3^ii^	97.89 (9)	O4^ii^—Mg1A—O4^xvii^	108.66 (2)
O7—Be1—O3^iii^	119.45 (7)	O4^viii^—Mg1A—O6	110.27 (2)
O3^i^—Be1—O3^iii^	97.89 (9)	O4^ii^—Mg1A—O6	110.27 (2)
O3^ii^—Be1—O3^iii^	97.89 (9)	O4^xvii^—Mg1A—O6	110.27 (2)
O7—Be1—Al1^iv^	138.88 (3)	O4^viii^—Mg1A—Al5^xix^	121.368 (4)
O3^i^—Be1—Al1^iv^	48.95 (5)	O4^ii^—Mg1A—Al5^xix^	121.369 (4)
O3^ii^—Be1—Al1^iv^	48.95 (5)	O4^xvii^—Mg1A—Al5^xix^	29.94 (2)
O3^iii^—Be1—Al1^iv^	101.67 (10)	O6—Mg1A—Al5^xix^	80.334 (11)
O7—Be1—Al1^v^	138.88 (3)	O4^viii^—Mg1A—Al5	121.368 (4)
O3^i^—Be1—Al1^v^	48.95 (5)	O4^ii^—Mg1A—Al5	29.94 (2)
O3^ii^—Be1—Al1^v^	101.67 (10)	O4^xvii^—Mg1A—Al5	121.368 (4)
O3^iii^—Be1—Al1^v^	48.95 (5)	O6—Mg1A—Al5	80.336 (11)
Al1^iv^—Be1—Al1^v^	69.44 (5)	Al5^xix^—Mg1A—Al5	117.240 (6)
O7—Be1—Al1^vi^	138.88 (3)	O4^viii^—Mg1A—Al5^xiii^	29.94 (2)
O3^i^—Be1—Al1^vi^	101.67 (10)	O4^ii^—Mg1A—Al5^xiii^	121.369 (4)
O3^ii^—Be1—Al1^vi^	48.95 (5)	O4^xvii^—Mg1A—Al5^xiii^	121.369 (4)
O3^iii^—Be1—Al1^vi^	48.95 (5)	O6—Mg1A—Al5^xiii^	80.334 (11)
Al1^iv^—Be1—Al1^vi^	69.44 (5)	Al5^xix^—Mg1A—Al5^xiii^	117.239 (6)
Al1^v^—Be1—Al1^vi^	69.44 (5)	Al5—Mg1A—Al5^xiii^	117.240 (6)
O3^vii^—Al1—O3^viii^	180.00 (4)	O4^viii^—Mg1A—Al3A^iv^	80.84 (2)
O3^vii^—Al1—O3^ix^	83.60 (4)	O4^ii^—Mg1A—Al3A^iv^	122.16 (2)
O3^viii^—Al1—O3^ix^	96.40 (4)	O4^xvii^—Mg1A—Al3A^iv^	122.16 (2)
O3^vii^—Al1—O3^x^	96.40 (4)	O6—Mg1A—Al3A^iv^	29.432 (6)
O3^viii^—Al1—O3^x^	83.60 (4)	Al5^xix^—Mg1A—Al3A^iv^	95.507 (12)
O3^ix^—Al1—O3^x^	180.00 (4)	Al5—Mg1A—Al3A^iv^	95.508 (12)
O3^vii^—Al1—O2	84.58 (3)	Al5^xiii^—Mg1A—Al3A^iv^	50.903 (10)
O3^viii^—Al1—O2	95.42 (3)	O4^viii^—Mg1A—Cr3B^iv^	80.84 (2)
O3^ix^—Al1—O2	84.58 (3)	O4^ii^—Mg1A—Cr3B^iv^	122.16 (2)
O3^x^—Al1—O2	95.42 (3)	O4^xvii^—Mg1A—Cr3B^iv^	122.16 (2)
O3^vii^—Al1—O2^xi^	95.42 (3)	O6—Mg1A—Cr3B^iv^	29.432 (6)
O3^viii^—Al1—O2^xi^	84.58 (3)	Al5^xix^—Mg1A—Cr3B^iv^	95.507 (12)
O3^ix^—Al1—O2^xi^	95.42 (3)	Al5—Mg1A—Cr3B^iv^	95.508 (12)
O3^x^—Al1—O2^xi^	84.58 (3)	Al5^xiii^—Mg1A—Cr3B^iv^	50.903 (10)
O2—Al1—O2^xi^	180.0	Al3A^iv^—Mg1A—Cr3B^iv^	0.000 (5)
O3^vii^—Al1—Be1^xii^	41.80 (2)	O4^viii^—Mg1A—Al3A^iii^	122.16 (2)
O3^viii^—Al1—Be1^xii^	138.20 (2)	O4^ii^—Mg1A—Al3A^iii^	80.84 (2)
O3^ix^—Al1—Be1^xii^	41.80 (2)	O4^xvii^—Mg1A—Al3A^iii^	122.16 (2)
O3^x^—Al1—Be1^xii^	138.20 (2)	O6—Mg1A—Al3A^iii^	29.432 (6)
O2—Al1—Be1^xii^	82.41 (4)	Al5^xix^—Mg1A—Al3A^iii^	95.508 (12)
O2^xi^—Al1—Be1^xii^	97.59 (4)	Al5—Mg1A—Al3A^iii^	50.904 (10)
O3^vii^—Al1—Be1^xiii^	138.20 (2)	Al5^xiii^—Mg1A—Al3A^iii^	95.508 (12)
O3^viii^—Al1—Be1^xiii^	41.80 (2)	Al3A^iv^—Mg1A—Al3A^iii^	50.371 (11)
O3^ix^—Al1—Be1^xiii^	138.20 (2)	Cr3B^iv^—Mg1A—Al3A^iii^	50.4
O3^x^—Al1—Be1^xiii^	41.80 (2)	O4^viii^—Mg1A—Cr3B^iii^	122.16 (2)
O2—Al1—Be1^xiii^	97.59 (4)	O4^ii^—Mg1A—Cr3B^iii^	80.84 (2)
O2^xi^—Al1—Be1^xiii^	82.41 (4)	O4^xvii^—Mg1A—Cr3B^iii^	122.16 (2)
Be1^xii^—Al1—Be1^xiii^	180.00 (7)	O6—Mg1A—Cr3B^iii^	29.432 (6)
O3^vii^—Al1—Al1^xiv^	139.043 (16)	Al5^xix^—Mg1A—Cr3B^iii^	95.508 (12)
O3^viii^—Al1—Al1^xiv^	40.957 (16)	Al5—Mg1A—Cr3B^iii^	50.904 (10)
O3^ix^—Al1—Al1^xiv^	95.088 (18)	Al5^xiii^—Mg1A—Cr3B^iii^	95.508 (12)
O3^x^—Al1—Al1^xiv^	84.912 (18)	Al3A^iv^—Mg1A—Cr3B^iii^	50.371 (11)
O2—Al1—Al1^xiv^	136.211 (19)	Cr3B^iv^—Mg1A—Cr3B^iii^	50.371 (11)
O2^xi^—Al1—Al1^xiv^	43.790 (19)	Al3A^iii^—Mg1A—Cr3B^iii^	0.000 (15)
Be1^xii^—Al1—Al1^xiv^	124.72 (3)	O5^viii^—Mg2A—O5^xxiv^	109.66 (2)
Be1^xiii^—Al1—Al1^xiv^	55.28 (3)	O5^viii^—Mg2A—O5^iii^	109.66 (2)
O3^vii^—Al1—Al1^xv^	40.957 (16)	O5^xxiv^—Mg2A—O5^iii^	109.66 (2)
O3^viii^—Al1—Al1^xv^	139.043 (16)	O5^viii^—Mg2A—O1	109.28 (2)
O3^ix^—Al1—Al1^xv^	84.912 (18)	O5^xxiv^—Mg2A—O1	109.28 (2)
O3^x^—Al1—Al1^xv^	95.088 (18)	O5^iii^—Mg2A—O1	109.28 (2)
O2—Al1—Al1^xv^	43.789 (19)	O5^viii^—Mg2A—Al5^viii^	121.522 (3)
O2^xi^—Al1—Al1^xv^	136.210 (19)	O5^xxiv^—Mg2A—Al5^viii^	29.45 (2)
Be1^xii^—Al1—Al1^xv^	55.28 (3)	O5^iii^—Mg2A—Al5^viii^	121.522 (3)
Be1^xiii^—Al1—Al1^xv^	124.72 (3)	O1—Mg2A—Al5^viii^	79.835 (12)
Al1^xiv^—Al1—Al1^xv^	180.0	O5^viii^—Mg2A—Al5^xiii^	29.45 (2)
O3^vii^—Al1—Al1^vi^	84.912 (18)	O5^xxiv^—Mg2A—Al5^xiii^	121.522 (3)
O3^viii^—Al1—Al1^vi^	95.088 (18)	O5^iii^—Mg2A—Al5^xiii^	121.522 (3)
O3^ix^—Al1—Al1^vi^	40.957 (16)	O1—Mg2A—Al5^xiii^	79.835 (12)
O3^x^—Al1—Al1^vi^	139.043 (16)	Al5^viii^—Mg2A—Al5^xiii^	116.954 (7)
O2—Al1—Al1^vi^	43.789 (19)	O5^viii^—Mg2A—Al5	121.521 (4)
O2^xi^—Al1—Al1^vi^	136.211 (19)	O5^xxiv^—Mg2A—Al5	121.521 (3)
Be1^xii^—Al1—Al1^vi^	55.28 (3)	O5^iii^—Mg2A—Al5	29.45 (2)
Be1^xiii^—Al1—Al1^vi^	124.72 (3)	O1—Mg2A—Al5	79.834 (12)
Al1^xiv^—Al1—Al1^vi^	120.0	Al5^viii^—Mg2A—Al5	116.954 (7)
Al1^xv^—Al1—Al1^vi^	60.0	Al5^xiii^—Mg2A—Al5	116.954 (7)
O3^vii^—Al1—Al1^xvi^	95.088 (18)	O5^viii^—Mg2A—Al3A^iv^	29.673 (12)
O3^viii^—Al1—Al1^xvi^	84.912 (18)	O5^xxiv^—Mg2A—Al3A^iv^	121.37 (3)
O3^ix^—Al1—Al1^xvi^	139.043 (16)	O5^iii^—Mg2A—Al3A^iv^	79.990 (15)
O3^x^—Al1—Al1^xvi^	40.957 (16)	O1—Mg2A—Al3A^iv^	121.802 (9)
O2—Al1—Al1^xvi^	136.211 (19)	Al5^viii^—Mg2A—Al3A^iv^	144.821 (12)
O2^xi^—Al1—Al1^xvi^	43.789 (19)	Al5^xiii^—Mg2A—Al3A^iv^	50.834 (9)
Be1^xii^—Al1—Al1^xvi^	124.72 (3)	Al5—Mg2A—Al3A^iv^	95.349 (7)
Be1^xiii^—Al1—Al1^xvi^	55.28 (3)	O5^viii^—Mg2A—Al3A^viii^	29.674 (12)
Al1^xiv^—Al1—Al1^xvi^	60.0	O5^xxiv^—Mg2A—Al3A^viii^	79.990 (15)
Al1^xv^—Al1—Al1^xvi^	120.0	O5^iii^—Mg2A—Al3A^viii^	121.37 (3)
Al1^vi^—Al1—Al1^xvi^	180.0	O1—Mg2A—Al3A^viii^	121.802 (9)
O2—Al2—O8^i^	109.22 (3)	Al5^viii^—Mg2A—Al3A^viii^	95.348 (7)
O2—Al2—O8^iv^	109.22 (3)	Al5^xiii^—Mg2A—Al3A^viii^	50.834 (8)
O8^i^—Al2—O8^iv^	109.72 (3)	Al5—Mg2A—Al3A^viii^	144.822 (12)
O2—Al2—O8^xvi^	109.22 (3)	Al3A^iv^—Mg2A—Al3A^viii^	50.270 (9)
O8^i^—Al2—O8^xvi^	109.72 (3)	O5^viii^—Mg2A—Al3A^xvi^	79.990 (15)
O8^iv^—Al2—O8^xvi^	109.72 (3)	O5^xxiv^—Mg2A—Al3A^xvi^	29.674 (12)
O6—Al3A—O5^xvii^	96.66 (3)	O5^iii^—Mg2A—Al3A^xvi^	121.37 (3)
O6—Al3A—O5	96.66 (3)	O1—Mg2A—Al3A^xvi^	121.802 (9)
O5^xvii^—Al3A—O5	82.22 (4)	Al5^viii^—Mg2A—Al3A^xvi^	50.834 (9)
O6—Al3A—O8^v^	83.72 (3)	Al5^xiii^—Mg2A—Al3A^xvi^	95.348 (7)
O5^xvii^—Al3A—O8^v^	175.23 (3)	Al5—Mg2A—Al3A^xvi^	144.822 (12)
O5—Al3A—O8^v^	93.01 (3)	Al3A^iv^—Mg2A—Al3A^xvi^	94.786 (12)
O6—Al3A—O8^xviii^	83.72 (3)	Al3A^viii^—Mg2A—Al3A^xvi^	50.318 (10)
O5^xvii^—Al3A—O8^xviii^	93.01 (3)	O5^viii^—Mg2A—Al3A^xxiv^	121.37 (3)
O5—Al3A—O8^xviii^	175.23 (3)	O5^xxiv^—Mg2A—Al3A^xxiv^	29.673 (12)
O8^v^—Al3A—O8^xviii^	91.76 (4)	O5^iii^—Mg2A—Al3A^xxiv^	79.990 (15)
O6—Al3A—O7^xix^	178.60 (4)	O1—Mg2A—Al3A^xxiv^	121.802 (9)
O5^xvii^—Al3A—O7^xix^	84.39 (3)	Al5^viii^—Mg2A—Al3A^xxiv^	50.834 (8)
O5—Al3A—O7^xix^	84.40 (3)	Al5^xiii^—Mg2A—Al3A^xxiv^	144.821 (12)
O8^v^—Al3A—O7^xix^	95.31 (3)	Al5—Mg2A—Al3A^xxiv^	95.349 (7)
O8^xviii^—Al3A—O7^xix^	95.31 (3)	Al3A^iv^—Mg2A—Al3A^xxiv^	116.397 (17)
O6—Al3A—Cr3B^xvii^	138.48 (2)	Al3A^viii^—Mg2A—Al3A^xxiv^	94.786 (12)
O5^xvii^—Al3A—Cr3B^xvii^	42.307 (16)	Al3A^xvi^—Mg2A—Al3A^xxiv^	50.270 (9)
O5—Al3A—Cr3B^xvii^	85.296 (19)	O5^viii^—Mg2A—Al3A^iii^	79.989 (15)
O8^v^—Al3A—Cr3B^xvii^	137.735 (18)	O5^xxiv^—Mg2A—Al3A^iii^	121.37 (3)
O8^xviii^—Al3A—Cr3B^xvii^	91.271 (18)	O5^iii^—Mg2A—Al3A^iii^	29.674 (12)
O7^xix^—Al3A—Cr3B^xvii^	42.450 (19)	O1—Mg2A—Al3A^iii^	121.802 (9)
O6—Al3A—Al3A^xvii^	138.48 (2)	Al5^viii^—Mg2A—Al3A^iii^	144.822 (12)
O5^xvii^—Al3A—Al3A^xvii^	42.307 (16)	Al5^xiii^—Mg2A—Al3A^iii^	95.349 (7)
O5—Al3A—Al3A^xvii^	85.296 (19)	Al5—Mg2A—Al3A^iii^	50.835 (9)
O8^v^—Al3A—Al3A^xvii^	137.735 (18)	Al3A^iv^—Mg2A—Al3A^iii^	50.317 (10)
O8^xviii^—Al3A—Al3A^xvii^	91.271 (18)	Al3A^viii^—Mg2A—Al3A^iii^	94.786 (12)
O7^xix^—Al3A—Al3A^xvii^	42.450 (19)	Al3A^xvi^—Mg2A—Al3A^iii^	116.397 (17)
Cr3B^xvii^—Al3A—Al3A^xvii^	0.0	Al3A^xxiv^—Mg2A—Al3A^iii^	94.786 (12)
O6—Al3A—Al3A^xx^	138.48 (2)	O3^xvii^—Mg3—O3^ii^	103.70 (3)
O5^xvii^—Al3A—Al3A^xx^	85.296 (19)	O3^xvii^—Mg3—O3^viii^	103.70 (3)
O5—Al3A—Al3A^xx^	42.308 (16)	O3^ii^—Mg3—O3^viii^	103.70 (3)
O8^v^—Al3A—Al3A^xx^	91.271 (18)	O3^xvii^—Mg3—O8^xvi^	160.59 (4)
O8^xviii^—Al3A—Al3A^xx^	137.735 (18)	O3^ii^—Mg3—O8^xvi^	88.06 (2)
O7^xix^—Al3A—Al3A^xx^	42.451 (19)	O3^viii^—Mg3—O8^xvi^	88.06 (2)
Cr3B^xvii^—Al3A—Al3A^xx^	60.0	O3^xvii^—Mg3—O8^v^	88.06 (2)
Al3A^xvii^—Al3A—Al3A^xx^	60.0	O3^ii^—Mg3—O8^v^	88.06 (2)
O6—Al3A—Cr3B^xx^	138.48 (2)	O3^viii^—Mg3—O8^v^	160.59 (4)
O5^xvii^—Al3A—Cr3B^xx^	85.296 (19)	O8^xvi^—Mg3—O8^v^	76.80 (3)
O5—Al3A—Cr3B^xx^	42.308 (16)	O3^xvii^—Mg3—O8^xviii^	88.064 (19)
O8^v^—Al3A—Cr3B^xx^	91.271 (18)	O3^ii^—Mg3—O8^xviii^	160.59 (4)
O8^xviii^—Al3A—Cr3B^xx^	137.735 (18)	O3^viii^—Mg3—O8^xviii^	88.06 (2)
O7^xix^—Al3A—Cr3B^xx^	42.451 (19)	O8^xvi^—Mg3—O8^xviii^	76.80 (3)
Cr3B^xvii^—Al3A—Cr3B^xx^	60.0	O8^v^—Mg3—O8^xviii^	76.80 (3)
Al3A^xvii^—Al3A—Cr3B^xx^	60.0	O3^xvii^—Mg3—Al3A	82.33 (2)
Al3A^xx^—Al3A—Cr3B^xx^	0.000 (14)	O3^ii^—Mg3—Al3A	126.66 (2)
O6—Al3A—Cr3B^iii^	41.52 (2)	O3^viii^—Mg3—Al3A	126.66 (2)
O5^xvii^—Al3A—Cr3B^iii^	137.691 (16)	O8^xvi^—Mg3—Al3A	78.26 (3)
O5—Al3A—Cr3B^iii^	94.703 (19)	O8^v^—Mg3—Al3A	38.697 (14)
O8^v^—Al3A—Cr3B^iii^	42.266 (18)	O8^xviii^—Mg3—Al3A	38.697 (14)
O8^xviii^—Al3A—Cr3B^iii^	88.730 (18)	O3^xvii^—Mg3—Al3A^iii^	126.66 (2)
O7^xix^—Al3A—Cr3B^iii^	137.552 (19)	O3^ii^—Mg3—Al3A^iii^	82.33 (2)
Cr3B^xvii^—Al3A—Cr3B^iii^	180.0	O3^viii^—Mg3—Al3A^iii^	126.66 (2)
Al3A^xvii^—Al3A—Cr3B^iii^	180.0	O8^xvi^—Mg3—Al3A^iii^	38.697 (14)
Al3A^xx^—Al3A—Cr3B^iii^	120.0	O8^v^—Mg3—Al3A^iii^	38.697 (14)
Cr3B^xx^—Al3A—Cr3B^iii^	120.0	O8^xviii^—Mg3—Al3A^iii^	78.26 (3)
O6—Al3A—Al3A^iii^	41.52 (2)	Al3A—Mg3—Al3A^iii^	55.352 (15)
O5^xvii^—Al3A—Al3A^iii^	137.691 (16)	O3^xvii^—Mg3—Cr3B^iii^	126.66 (2)
O5—Al3A—Al3A^iii^	94.703 (19)	O3^ii^—Mg3—Cr3B^iii^	82.33 (2)
O8^v^—Al3A—Al3A^iii^	42.266 (18)	O3^viii^—Mg3—Cr3B^iii^	126.66 (2)
O8^xviii^—Al3A—Al3A^iii^	88.730 (18)	O8^xvi^—Mg3—Cr3B^iii^	38.697 (14)
O7^xix^—Al3A—Al3A^iii^	137.552 (19)	O8^v^—Mg3—Cr3B^iii^	38.697 (14)
Cr3B^xvii^—Al3A—Al3A^iii^	180.0	O8^xviii^—Mg3—Cr3B^iii^	78.26 (3)
Al3A^xvii^—Al3A—Al3A^iii^	180.0	Al3A—Mg3—Cr3B^iii^	55.4
Al3A^xx^—Al3A—Al3A^iii^	120.0	Al3A^iii^—Mg3—Cr3B^iii^	0.000 (16)
Cr3B^xx^—Al3A—Al3A^iii^	120.0	O3^xvii^—Mg3—Al3A^iv^	126.66 (2)
Cr3B^iii^—Al3A—Al3A^iii^	0.0	O3^ii^—Mg3—Al3A^iv^	126.66 (2)
O1^xxi^—Al4A—O1	180.0	O3^viii^—Mg3—Al3A^iv^	82.33 (2)
O1^xxi^—Al4A—O4^xxii^	96.18 (3)	O8^xvi^—Mg3—Al3A^iv^	38.697 (14)
O1—Al4A—O4^xxii^	83.82 (3)	O8^v^—Mg3—Al3A^iv^	78.26 (3)
O1^xxi^—Al4A—O4^x^	83.82 (3)	O8^xviii^—Mg3—Al3A^iv^	38.697 (14)
O1—Al4A—O4^x^	96.18 (3)	Al3A—Mg3—Al3A^iv^	55.352 (15)
O4^xxii^—Al4A—O4^x^	180.00 (3)	Al3A^iii^—Mg3—Al3A^iv^	55.351 (15)
O1^xxi^—Al4A—O4^xxiii^	96.18 (3)	Cr3B^iii^—Mg3—Al3A^iv^	55.4
O1—Al4A—O4^xxiii^	83.82 (3)	O3^xvii^—Mg3—Cr3B^iv^	126.66 (2)
O4^xxii^—Al4A—O4^xxiii^	83.33 (4)	O3^ii^—Mg3—Cr3B^iv^	126.66 (2)
O4^x^—Al4A—O4^xxiii^	96.67 (4)	O3^viii^—Mg3—Cr3B^iv^	82.33 (2)
O1^xxi^—Al4A—O4^viii^	83.82 (3)	O8^xvi^—Mg3—Cr3B^iv^	38.697 (14)
O1—Al4A—O4^viii^	96.18 (3)	O8^v^—Mg3—Cr3B^iv^	78.26 (3)
O4^xxii^—Al4A—O4^viii^	96.67 (4)	O8^xviii^—Mg3—Cr3B^iv^	38.697 (14)
O4^x^—Al4A—O4^viii^	83.33 (4)	Al3A—Mg3—Cr3B^iv^	55.4
O4^xxiii^—Al4A—O4^viii^	180.0	Al3A^iii^—Mg3—Cr3B^iv^	55.351 (15)
O1^xxi^—Al4A—Al4A^xiv^	42.07 (2)	Cr3B^iii^—Mg3—Cr3B^iv^	55.351 (15)
O1—Al4A—Al4A^xiv^	137.93 (2)	Al3A^iv^—Mg3—Cr3B^iv^	0.000 (6)
O4^xxii^—Al4A—Al4A^xiv^	94.432 (19)	Cr4B^xv^—O1—Al4A^xv^	0.0
O4^x^—Al4A—Al4A^xiv^	85.568 (19)	Cr4B^xv^—O1—Al4A	95.9
O4^xxiii^—Al4A—Al4A^xiv^	137.904 (15)	Al4A^xv^—O1—Al4A	95.86 (4)
O4^viii^—Al4A—Al4A^xiv^	42.096 (15)	Cr4B^xv^—O1—Cr4B^vi^	95.86 (4)
O1^xxi^—Al4A—Al4A^xv^	137.93 (2)	Al4A^xv^—O1—Cr4B^vi^	95.86 (4)
O1—Al4A—Al4A^xv^	42.07 (2)	Al4A—O1—Cr4B^vi^	95.9
O4^xxii^—Al4A—Al4A^xv^	85.568 (19)	Cr4B^xv^—O1—Al4A^vi^	95.9
O4^x^—Al4A—Al4A^xv^	94.432 (19)	Al4A^xv^—O1—Al4A^vi^	95.86 (4)
O4^xxiii^—Al4A—Al4A^xv^	42.096 (15)	Al4A—O1—Al4A^vi^	95.86 (4)
O4^viii^—Al4A—Al4A^xv^	137.904 (15)	Cr4B^vi^—O1—Al4A^vi^	0.0
Al4A^xiv^—Al4A—Al4A^xv^	180.0	Cr4B^xv^—O1—Mg2A	121.00 (3)
O1^xxi^—Al4A—Cr4B^xiv^	42.07 (2)	Al4A^xv^—O1—Mg2A	121.00 (3)
O1—Al4A—Cr4B^xiv^	137.93 (2)	Al4A—O1—Mg2A	121.00 (3)
O4^xxii^—Al4A—Cr4B^xiv^	94.432 (19)	Cr4B^vi^—O1—Mg2A	121.00 (3)
O4^x^—Al4A—Cr4B^xiv^	85.568 (19)	Al4A^vi^—O1—Mg2A	121.00 (3)
O4^xxiii^—Al4A—Cr4B^xiv^	137.904 (15)	Al2—O2—Al1	123.53 (3)
O4^viii^—Al4A—Cr4B^xiv^	42.096 (15)	Al2—O2—Al1^xv^	123.53 (3)
Al4A^xiv^—Al4A—Cr4B^xiv^	0.0	Al1—O2—Al1^xv^	92.42 (4)
Al4A^xv^—Al4A—Cr4B^xiv^	180.0	Al2—O2—Al1^vi^	123.53 (3)
O1^xxi^—Al4A—Cr4B^xv^	137.93 (2)	Al1—O2—Al1^vi^	92.42 (4)
O1—Al4A—Cr4B^xv^	42.07 (2)	Al1^xv^—O2—Al1^vi^	92.42 (4)
O4^xxii^—Al4A—Cr4B^xv^	85.568 (19)	Be1^xix^—O3—Al1^xviii^	89.25 (5)
O4^x^—Al4A—Cr4B^xv^	94.432 (19)	Be1^xix^—O3—Al1^xxv^	89.25 (5)
O4^xxiii^—Al4A—Cr4B^xv^	42.096 (15)	Al1^xviii^—O3—Al1^xxv^	98.09 (3)
O4^viii^—Al4A—Cr4B^xv^	137.904 (15)	Be1^xix^—O3—Mg3^xxv^	125.79 (8)
Al4A^xiv^—Al4A—Cr4B^xv^	180.0	Al1^xviii^—O3—Mg3^xxv^	122.63 (2)
Al4A^xv^—Al4A—Cr4B^xv^	0.0	Al1^xxv^—O3—Mg3^xxv^	122.63 (2)
Cr4B^xiv^—Al4A—Cr4B^xv^	180.0	Cr4B^xxv^—O4—Al4A^xxv^	0.0
O1^xxi^—Al4A—Al4A^vi^	137.93 (2)	Cr4B^xxv^—O4—Cr4B^xviii^	95.81 (3)
O1—Al4A—Al4A^vi^	42.07 (2)	Al4A^xxv^—O4—Cr4B^xviii^	95.81 (3)
O4^xxii^—Al4A—Al4A^vi^	42.096 (15)	Cr4B^xxv^—O4—Al4A^xviii^	95.8
O4^x^—Al4A—Al4A^vi^	137.904 (15)	Al4A^xxv^—O4—Al4A^xviii^	95.81 (3)
O4^xxiii^—Al4A—Al4A^vi^	85.569 (18)	Cr4B^xviii^—O4—Al4A^xviii^	0.0
O4^viii^—Al4A—Al4A^vi^	94.431 (18)	Cr4B^xxv^—O4—Al5^xix^	96.34 (3)
Al4A^xiv^—Al4A—Al4A^vi^	120.0	Al4A^xxv^—O4—Al5^xix^	96.34 (3)
Al4A^xv^—Al4A—Al4A^vi^	60.0	Cr4B^xviii^—O4—Al5^xix^	96.34 (3)
Cr4B^xiv^—Al4A—Al4A^vi^	120.0	Al4A^xviii^—O4—Al5^xix^	96.34 (3)
Cr4B^xv^—Al4A—Al4A^vi^	60.0	Cr4B^xxv^—O4—Cr1B^xxv^	121.23 (2)
O1^xxi^—Al4A—Cr4B^vi^	137.93 (2)	Al4A^xxv^—O4—Cr1B^xxv^	121.23 (2)
O1—Al4A—Cr4B^vi^	42.07 (2)	Cr4B^xviii^—O4—Cr1B^xxv^	121.23 (2)
O4^xxii^—Al4A—Cr4B^vi^	42.096 (15)	Al4A^xviii^—O4—Cr1B^xxv^	121.23 (2)
O4^x^—Al4A—Cr4B^vi^	137.904 (15)	Al5^xix^—O4—Cr1B^xxv^	119.86 (4)
O4^xxiii^—Al4A—Cr4B^vi^	85.569 (18)	Cr4B^xxv^—O4—Mg1A^xxv^	121.23 (2)
O4^viii^—Al4A—Cr4B^vi^	94.431 (18)	Al4A^xxv^—O4—Mg1A^xxv^	121.23 (2)
Al4A^xiv^—Al4A—Cr4B^vi^	120.0	Cr4B^xviii^—O4—Mg1A^xxv^	121.23 (2)
Al4A^xv^—Al4A—Cr4B^vi^	60.0	Al4A^xviii^—O4—Mg1A^xxv^	121.23 (2)
Cr4B^xiv^—Al4A—Cr4B^vi^	120.0	Al5^xix^—O4—Mg1A^xxv^	119.86 (4)
Cr4B^xv^—Al4A—Cr4B^vi^	60.0	Cr1B^xxv^—O4—Mg1A^xxv^	0.0
Al4A^vi^—Al4A—Cr4B^vi^	0.0	Al5^xix^—O5—Cr3B^xx^	97.03 (3)
O5^iii^—Al5—O5^ii^	83.03 (3)	Al5^xix^—O5—Al3A^xx^	97.03 (3)
O5^iii^—Al5—O5^i^	83.03 (3)	Cr3B^xx^—O5—Al3A^xx^	0.0
O5^ii^—Al5—O5^i^	83.02 (3)	Al5^xix^—O5—Al3A	97.03 (3)
O5^iii^—Al5—O4^iii^	96.83 (2)	Cr3B^xx^—O5—Al3A	95.4
O5^ii^—Al5—O4^iii^	179.81 (3)	Al3A^xx^—O5—Al3A	95.38 (3)
O5^i^—Al5—O4^iii^	96.83 (2)	Al5^xix^—O5—Cr2B^xxv^	120.65 (4)
O5^iii^—Al5—O4^ii^	96.83 (2)	Cr3B^xx^—O5—Cr2B^xxv^	120.46 (2)
O5^ii^—Al5—O4^ii^	96.83 (2)	Al3A^xx^—O5—Cr2B^xxv^	120.46 (2)
O5^i^—Al5—O4^ii^	179.81 (3)	Al3A—O5—Cr2B^xxv^	120.46 (2)
O4^iii^—Al5—O4^ii^	83.31 (3)	Al5^xix^—O5—Mg2A^xxv^	120.65 (4)
O5^iii^—Al5—O4^i^	179.81 (3)	Cr3B^xx^—O5—Mg2A^xxv^	120.46 (2)
O5^ii^—Al5—O4^i^	96.83 (2)	Al3A^xx^—O5—Mg2A^xxv^	120.46 (2)
O5^i^—Al5—O4^i^	96.83 (2)	Al3A—O5—Mg2A^xxv^	120.46 (2)
O4^iii^—Al5—O4^i^	83.31 (3)	Cr2B^xxv^—O5—Mg2A^xxv^	0.0
O4^ii^—Al5—O4^i^	83.31 (3)	Cr3B^iii^—O6—Al3A^iii^	0.0
O5^iii^—Al5—Cr4B^v^	138.301 (15)	Cr3B^iii^—O6—Cr3B^iv^	96.97 (4)
O5^ii^—Al5—Cr4B^v^	138.300 (15)	Al3A^iii^—O6—Cr3B^iv^	96.97 (4)
O5^i^—Al5—Cr4B^v^	94.97 (2)	Cr3B^iii^—O6—Al3A^iv^	97.0
O4^iii^—Al5—Cr4B^v^	41.825 (15)	Al3A^iii^—O6—Al3A^iv^	96.97 (4)
O4^ii^—Al5—Cr4B^v^	85.22 (2)	Cr3B^iv^—O6—Al3A^iv^	0.0
O4^i^—Al5—Cr4B^v^	41.826 (15)	Cr3B^iii^—O6—Al3A	97.0
O5^iii^—Al5—Al4A^v^	138.301 (15)	Al3A^iii^—O6—Al3A	96.97 (4)
O5^ii^—Al5—Al4A^v^	138.300 (15)	Cr3B^iv^—O6—Al3A	97.0
O5^i^—Al5—Al4A^v^	94.97 (2)	Al3A^iv^—O6—Al3A	96.97 (4)
O4^iii^—Al5—Al4A^v^	41.825 (15)	Cr3B^iii^—O6—Mg1A	120.16 (3)
O4^ii^—Al5—Al4A^v^	85.22 (2)	Al3A^iii^—O6—Mg1A	120.16 (3)
O4^i^—Al5—Al4A^v^	41.826 (15)	Cr3B^iv^—O6—Mg1A	120.16 (3)
Cr4B^v^—Al5—Al4A^v^	0.0	Al3A^iv^—O6—Mg1A	120.16 (3)
O5^iii^—Al5—Cr4B^iv^	138.301 (15)	Al3A—O6—Mg1A	120.16 (3)
O5^ii^—Al5—Cr4B^iv^	94.97 (2)	Be1—O7—Cr3B^ii^	121.57 (3)
O5^i^—Al5—Cr4B^iv^	138.300 (15)	Be1—O7—Al3A^ii^	121.57 (3)
O4^iii^—Al5—Cr4B^iv^	85.22 (2)	Cr3B^ii^—O7—Al3A^ii^	0.0
O4^ii^—Al5—Cr4B^iv^	41.825 (15)	Be1—O7—Al3A^i^	121.57 (3)
O4^i^—Al5—Cr4B^iv^	41.826 (15)	Cr3B^ii^—O7—Al3A^i^	95.1
Cr4B^v^—Al5—Cr4B^iv^	59.719 (10)	Al3A^ii^—O7—Al3A^i^	95.10 (4)
Al4A^v^—Al5—Cr4B^iv^	59.719 (10)	Be1—O7—Cr3B^i^	121.57 (3)
O5^iii^—Al5—Al4A^iv^	138.301 (15)	Cr3B^ii^—O7—Cr3B^i^	95.10 (4)
O5^ii^—Al5—Al4A^iv^	94.97 (2)	Al3A^ii^—O7—Cr3B^i^	95.10 (4)
O5^i^—Al5—Al4A^iv^	138.300 (15)	Al3A^i^—O7—Cr3B^i^	0.000 (18)
O4^iii^—Al5—Al4A^iv^	85.22 (2)	Be1—O7—Cr3B^iii^	121.57 (3)
O4^ii^—Al5—Al4A^iv^	41.825 (15)	Cr3B^ii^—O7—Cr3B^iii^	95.10 (4)
O4^i^—Al5—Al4A^iv^	41.826 (15)	Al3A^ii^—O7—Cr3B^iii^	95.10 (4)
Cr4B^v^—Al5—Al4A^iv^	59.7	Al3A^i^—O7—Cr3B^iii^	95.10 (4)
Al4A^v^—Al5—Al4A^iv^	59.719 (10)	Cr3B^i^—O7—Cr3B^iii^	95.10 (4)
Cr4B^iv^—Al5—Al4A^iv^	0.0	Be1—O7—Al3A^iii^	121.57 (3)
O5^iii^—Al5—Cr4B^vi^	94.97 (2)	Cr3B^ii^—O7—Al3A^iii^	95.1
O5^ii^—Al5—Cr4B^vi^	138.301 (15)	Al3A^ii^—O7—Al3A^iii^	95.10 (4)
O5^i^—Al5—Cr4B^vi^	138.301 (15)	Al3A^i^—O7—Al3A^iii^	95.10 (4)
O4^iii^—Al5—Cr4B^vi^	41.826 (15)	Cr3B^i^—O7—Al3A^iii^	95.1
O4^ii^—Al5—Cr4B^vi^	41.826 (15)	Cr3B^iii^—O7—Al3A^iii^	0.0
O4^i^—Al5—Cr4B^vi^	85.22 (2)	Al2^xix^—O8—Cr3B^x^	123.84 (2)
Cr4B^v^—Al5—Cr4B^vi^	59.719 (10)	Al2^xix^—O8—Al3A^x^	123.84 (2)
Al4A^v^—Al5—Cr4B^vi^	59.719 (10)	Cr3B^x^—O8—Al3A^x^	0.0
Cr4B^iv^—Al5—Cr4B^vi^	59.719 (10)	Al2^xix^—O8—Al3A^xiii^	123.84 (2)
Al4A^iv^—Al5—Cr4B^vi^	59.719 (10)	Cr3B^x^—O8—Al3A^xiii^	95.5
O5^iii^—Al5—Al4A^vi^	94.97 (2)	Al3A^x^—O8—Al3A^xiii^	95.47 (4)
O5^ii^—Al5—Al4A^vi^	138.301 (15)	Al2^xix^—O8—Cr3B^xiii^	123.84 (2)
O5^i^—Al5—Al4A^vi^	138.301 (15)	Cr3B^x^—O8—Cr3B^xiii^	95.47 (4)
O4^iii^—Al5—Al4A^vi^	41.826 (15)	Al3A^x^—O8—Cr3B^xiii^	95.47 (4)
O4^ii^—Al5—Al4A^vi^	41.826 (15)	Al3A^xiii^—O8—Cr3B^xiii^	0.000 (17)
O4^i^—Al5—Al4A^vi^	85.22 (2)	Al2^xix^—O8—Mg3^xiii^	116.60 (4)
Cr4B^v^—Al5—Al4A^vi^	59.7	Cr3B^x^—O8—Mg3^xiii^	95.04 (3)
Al4A^v^—Al5—Al4A^vi^	59.719 (10)	Al3A^x^—O8—Mg3^xiii^	95.04 (3)
Cr4B^iv^—Al5—Al4A^vi^	59.7	Al3A^xiii^—O8—Mg3^xiii^	95.04 (3)
Al4A^iv^—Al5—Al4A^vi^	59.719 (10)	Cr3B^xiii^—O8—Mg3^xiii^	95.04 (3)
Cr4B^vi^—Al5—Al4A^vi^	0.0		

Symmetry codes: (i) *x*+1, *y*+1, *z*; (ii) −*y*, *x*−*y*+1, *z*; (iii) −*x*+*y*, −*x*, *z*; (iv) −*y*, *x*−*y*, *z*; (v) *x*, *y*+1, *z*; (vi) −*x*+*y*+1, −*x*+1, *z*; (vii) −*x*, −*y*, −*z*; (viii) *x*+1, *y*, *z*; (ix) *x*−*y*+1, *x*+1, −*z*; (x) −*x*+*y*, −*x*−1, *z*; (xi) −*x*+1, −*y*, −*z*; (xii) −*x*+1, −*y*+1, −*z*; (xiii) *x*, *y*−1, *z*; (xiv) −*y*, *x*−*y*−1, *z*; (xv) −*y*+1, *x*−*y*, *z*; (xvi) −*x*+*y*+1, −*x*, *z*; (xvii) −*x*+*y*−1, −*x*−1, *z*; (xviii) −*y*−1, *x*−*y*−1, *z*; (xix) *x*−1, *y*−1, *z*; (xx) −*y*−1, *x*−*y*, *z*; (xxi) −*x*+1, −*y*, −*z*+1; (xxii) *x*−*y*+1, *x*+1, −*z*+1; (xxiii) −*x*, −*y*, −*z*+1; (xxiv) −*y*+1, *x*−*y*+1, *z*; (xxv) *x*−1, *y*, *z*.
